# Biogeographical Differences in the Influence of Maternal Microbial Sources on the Early Successional Development of the Bovine Neonatal Gastrointestinal tract

**DOI:** 10.1038/s41598-018-21440-8

**Published:** 2018-02-16

**Authors:** Carl J. Yeoman, Suzanne L. Ishaq, Elena Bichi, Sarah K. Olivo, James Lowe, Brian M. Aldridge

**Affiliations:** 1 Montana State University, Department of Animal and Range Science, Bozeman, MT USA; 2 Integrated Food Animal Systems, College of Veterinary Medicine, University of Illinois, Urbana-Champaign, IL USA

## Abstract

The impact of maternal microbial influences on the early choreography of the neonatal calf microbiome were investigated. Luminal content and mucosal scraping samples were collected from ten locations in the calf gastrointestinal tract (GIT) over the first 21 days of life, along with postpartum maternal colostrum, udder skin, and vaginal scrapings. Microbiota were found to vary by anatomical location, between the lumen and mucosa at each GIT location, and differentially enriched for maternal vaginal, skin, and colostral microbiota. Most calf sample sites exhibited a gradual increase in α-diversity over the 21 days beginning the first few days after birth. The relative abundance of Firmicutes was greater in the proximal GIT, while Bacteroidetes were greater in the distal GIT. Proteobacteria exhibited greater relative abundances in mucosal scrapings relative to luminal content. Forty-six percent of calf luminal microbes and 41% of mucosal microbes were observed in at-least one maternal source, with the majority being shared with microbes on the skin of the udder. The vaginal microbiota were found to harbor and uniquely share many common and well-described fibrolytic rumen bacteria, as well as methanogenic archaea, potentially indicating a role for the vagina in populating the developing rumen and reticulum with microbes important to the nutrition of the adult animal.

## Introduction

The microbiota of the GIT are of critical importance to the nutritive processes, health, and development of all known mammals, including cattle and other ruminants^1–3^. Colonization of the ruminant gastrointestinal tract (GIT) begins during birth and progresses through until a climactic community establishes^4–8^. Once this climax community is reached, the GIT microbiome is generally considered to be stable, excepting for changes in diet, physiological state, or health status^9–13^. Adult microbial diversity profiles do, however, differ along the GIT exhibiting biospatial circumscriptions that separate anatomical locations^14–16^. Similarly, there are also differences known to exist between microbes occupying the lumen, and those found associated with the mucosal-epithelia of the GIT^15,17^. These differences are likely due to the varying niches imparted by localized variations in nutrient profiles, pH, transit rates, host physiology, immune cell populations, and the differing interactions between host-epithelia and symbiotic bacteria^18–20^. For example, pH varies through the rumen GIT being lowest in the abomasum (~pH 2–4) and highest in the distal GIT (~pH 6)^21^; The rate at which digesta transits through the GIT is affected by diet, but is generally faster in the small intestine than the abomasum^22^ and slowest in the rumen and large intestine (cecum & colon)^23^; while a steep oxygen gradient runs through the epithelium to the hypoxic lumen^24^. Each of these factors refine the colonization potential at each site limiting occupation to the microbes for which these collection of conditions fall within their fundamental niche leading to described and hypothesized impacts on the microbial composition^25–27^. How each location of the ruminant GIT is seeded and the ecological dynamics underpinning their succession remains to be described. Findings from studies on human neonatal colonization^28^ have led those studying cattle to hypothesize that the earliest exposure of neonatal calves to microorganisms also occurs during parturition, when the calf is exposed to microbes occupying the dam’s vagina^29^. The calf may subsequently be exposed to microbes residing on the dam’s skin^28^, and recent evidence of a colostrum microbiota have led to the hypothesis that it may also provide an early source of microbes to the neonatal GIT^30–32^. Consistently, the DNA of common GIT microbes have been reported at low relative abundances in breast milk of cattle, goats, and humans^30,32–37^. Important microbial groups and critical nutritive functions such as hemicellulolytic (3 d), pectinolytic (3 d), cellulolytic (2–3 w), and methanogenic (1–3d) activities have been shown to emerge early in the rumen^4,7,38^. However, the successional development of the rumen appears to be far more protracted with substantial dynamism initially before settling on a climactic adult-like state^8^, similar to what has been described in humans^39^. For ruminants, these changes may partially reflect dietary transitions from colostrum to milk or milk replacer, and then to a progression of solid diets given in production settings to adapt the animal to an adult feeding regimen^17^. Successional changes in the GIT biochemistry brought about by these dietary transitions, as well as each colonizing species’ metabolic activities are also likely to impact community membership^40,41^. It is also likely that stochastic processes consistent with the unified neutral theory of biodiversity^42^ are involved, though the degree and duration are unclear.

Because the actual influence of maternal sources of microbes to the early succession of the calf GIT has neither been experimentally determined nor quantified, we set out to determine the relationships among the microbiota of the dam’s vagina, skin, and colostrum to the early successional development of the calf microbiome. The dam’s udder was used as our representative skin microbiota because under typical conditions it is more likely to directly interact with the calf’s GIT than other skin surfaces. Additionally, because colostrum could potentially contain autocthonous microbiota from the mammary alveolus or intralobular duct, along with auto- or allocthonous microbiota picked up from the teat or surrounding skin (potentially even penetrating the inner ducts or orifice of the teat), we wished to compare the outer skin of the udder to colostrum.

## Methods

### Animals and Sample Collection

All experimental procedures were performed at Fair Oaks Farms (Fair Oaks, IN) and University of Illinois Veterinary Medicine Research Farm (Urbana-Champaign, IL) under protocols approved by the Institutional Animal Care and Use Committee (IACUC) of the University of Illinois (protocol #13096). Twelve healthy male Holstein calves were separated from their dam within 15 minutes of birth, dried off with a hair dryer and had their navel disinfected with iodine solution. No vaccination was given.

Calves were recruited to the study if their dam fulfilled the following criteria: 1) 2^nd^ lactation cow, 2) single calf, 3) colostrum quality assessed by a refractometer (Misco® DD-1) >20° Brix, corresponding at a minimum of 50 mg/L of IgG, 4) colostrum produced was at least 4 L. After birth, each calf received 2 liters of colostrum aseptically collected from the respective dam within 1 hour. After first feeding, colostrum was kept refrigerated and warmed up before administration. Calves were offered first milking colostrum again on the second feeding at 4 hours after birth. Calves were subsequently fed three times a day with a non-medicated milk replacer (Agrimaster®) for the remainder of the experiment and had access to fresh water *ad libitum*.

All calves were monitored, and fed separately in individual 10′ × 10′ indoor pens of the same barn. Pens were bedded with clean sawdust and straw. Only 2–4 calves were present in the barn at any one time and pens were separated from one another by ≥10 ft. Dams were not cohoused, nor had ever previously been housed in the same barn as calves. Milk replacer intake and health checks were recorded daily. Health checks included body temperature, animal behavior, respiration, and fecal score (calf health scoring chart, School of Veterinary Medicine, University of Wisconsin-Madison). Body weight was monitored every other day in order to adjust milk replacer intake accordingly (feeding rate: 10% of body weight). Extruded fecal samples were carefully collected from calves to minimize potential contamination at days 0, 1, 2, 3, 4, 5, 7, 14, and 21 after colostrum administration.

Three calves were euthanized at each of age 1, 3, 7 and 21 days for necropsy, and luminal digesta and mucosal scraping samples were collected from the pharynx, rumen, reticulum, omasum, abomasum, duodenum, jejunum (proximal, medial, and distal), ileum, and colon. Samples were also collected from the dam’s colostrum, outer udder skin, and vaginal scrapings immediately at calving. After collection, all samples were immediately frozen in liquid nitrogen and kept at −80 °C until analysis.

### Sequencing

Samples were processed for DNA using the PowerSoil 96-well DNA Isolation Kit (MoBio Laboratories, Inc., Carlsbad, CA) strictly adhering to the manufacturers instructions. The V3-V4 region of the 16S rRNA gene was PCR-amplified using the KAPA HotStart PCR Kit (Kapa Biosystems, Wilmington, MA): 10 µL Kappa HotStart Mastermix, 6 µL molecular-grade water, 1 µL of each forward and reverse primer, and 2 µL sample DNA. The Hot Start protocol was as follows: 95 °C for 3 min; 5 cycles of denaturation at 98 °C for 20 sec, annealing at 52 °C for 30 sec, elongation at 72 °C for 45 sec; 25 cycles of denaturation at 98 °C for 20 sec, annealing at 60 °C for 30 sec, elongation at 72 °C for 45 sec; followed by storage at 4 °C. Primers amplified the V3-V4 region of the microbial 16S rRNA gene, and included the MiSeq adaptors (A for forward, B for reverse), the sample index/barcodes, the two-nucleotide linker, and the primers 341 F 5′-CCTACGGGAGGCAGCAG-3′and 806 R 5′-GGACTACHVGGGTWTCTAAT-3′.

PCR amplicon concentration and quality was checked on a TapeStation Bioanalyzer (Agilent Technologies, Santa Clara, CA) using the D1000 Screentapes with 3 µl of D1000 buffer and 1 μl of PCR product in each tube. Samples were then diluted with molecular water and pooled at equimolar concentrations of amplicon. The pooled amplicons were gel purified from a 1.5% agarose gel using the QIAquick gel extraction kit (QIAGEN, Valencia, CA) according to kit instructions. Sequencing was performed on an Illumina MiSeq (Illumina, San Diego, CA) using a 2 × 251 nt sequencing approach with a 500 cycle v2 kit. A no-template (molecular grade water) negative control and a custom 32 member mock community positive control were also processed and sequenced. Resulting FASTQ files, and can be found in the NCBI Sequence Read Archive (SRA) under BioProject PRJNA314799.

### DNA analysis

Samples were demultiplexed using CASAVA software v1.8.2 (Illumina, San Diego, CA). Paired-end 16S rRNA sequences for each sample were assembled into contiguous sequences (contigs), using PANDAseq.^43^ with default assembly parameters. Assembled sequences were then curated using in-house scripts and mothur v.1.35^44^ as follows: First, sequences were trimmed after any homopolymer >8 nt long using an in-house script. This was justified as homopolymer length is not directly tied to whole sequence error rate in Illumina MiSeq data^45^. Mothur was then used to remove sequences which were <400 nt or >580 nt, or which contained any ambiguous bases (N). Sequences were aligned using mothur to the Silva v119 reference database, which contains bacterial and archaeal 16S rRNA gene sequences using the Wang algorithm^46^. Chimeras were identified and removed using the mother-integrated version of UCHIME^47^, and sequences were pre-clustered by 2 base differences as previously described^48^. Sequences were classified using mother-implemented RDP classifier using the Silva v119 taxonomy database at 80% confidence cutoff^46^. Taxonomy was used to bin sequences into Operational Taxonomic Units (OTUs) at the genus-level, which is the lowest limit of taxonomic resolution available from the Silva taxonomy. Three OTUs detected as contaminant in blank and mock controls (classified as *Brevundimonas*, *Comamonas*, and *Ralstonia*) were removed prior to analyses.

Sequences were subsampled to the lowest number (min. 2500) of reads for each statistical analysis. The diversity measures ACE, CHAO, Inverse Simpson, Good’s Coverage, Shannon-Weiner diversity, and UniFrac^49–53^ values are presented as group mean. Linear Discriminant Analysis was run to detect discriminatory OTUs, using Wilcoxon Rank Test to determine significance. The R statistical package ggplot2 was used to create heatmaps^54^. PRIMER ver. 6^55^ was used to determine Bray-Curtis Dissimilarities among samples, create a Non-metric Multi-Dimensional Scaling (nMDS), perform ANOSIM and PERMANOVA calculations, and determine Ewans-Caswell statistics for assessing the deviations from the assumptions of neutrality^42^. One way ANOVA was used to test for significant differences among taxa, in the Bray-Curtis similarities (1 – dissimilarity) between maternal and calf GIT samples, and between sample types for Ewans-Caswell V statistics. All statistics were corrected for multiple tests using the Benjamini-Hochberg FDR approach where necessary.

## Results

### General Features of the Dataset

A total of 2,511,755 high-quality sequences were clustered to give a total of 1,213 OTUs. Among OTUs, 75% of taxonomic classifications were supported at the genus level and Good’s estimates of coverage indicated we had captured sequences from the majority of OTUs present (Good’s > 0.96; Table 1). Although our primers were designed to capture both bacteria and archaea, the majority of OTUs were bacterial, deriving from a total of 326 bacterial families and 925 bacterial genera. A smaller number of archaeal sequences were identified and were classified into five families, with the majority of these sequences belonging to the genus *Methanobrevibacter*.

**Table 1:** Mean diversity statistics by sample groups. Any groups which did not have adequate sequences/sample for statistical comparison were not included and are marked with “−”, as opposed to “n/a” designating that the sample type was not collected for that anatomical location.

Group Mean	CHAO	ACE	Observed OTUs	Good’s Coverage	Inverse Simpson	CHAO	ACE	Observed OTUs	Good’s Coverage	Inverse Simpson
**Maternal**										
Colostrum (1st)	222	250	163	0.979	24.81					
Colostrum (2nd)	243	308	162	0.976	22.99					
Udder skin scraping	268	276	217	0.978	43.86					
Vaginal scraping	213	217	152	0.981	22.41					
**Calf**										
	**Lumen**					**Mucosa**				
Pharynx Day 1	n/a					95	160	38	0.992	2.54
Day 3	n/a					86	96	58	0.992	5.88
Day 7	n/a					91	110	55	0.991	3.67
Day 21	n/a					83	88	54	0.992	5.43
Rumen Day 1	49	56	37	0.995	4.94	108	107	96	0.993	12.85
Day 3	63	66	51	0.995	6.60	94	95	82	0.993	11.21
Day 7	60	65	50	0.995	5.81	73	63	46	0.994	4.68
Day 21	77	88	55	0.993	6.90	77	79	63	0.993	5.35
Reticulum Day 1	—	—	—	—	—	75	111	48	0.992	4.88
Day 3	46	43	32	0.996	5.46	66	87	52	0.995	5.87
Day 7	41	44	35	0.996	4.38	52	55	44	0.996	6.25
Day 21	66	77	49	0.995	6.56	77	87	54	0.993	5.50
Omasum Day 1	47	64	31	0.995	4.71	56	75	37	0.994	4.21
Day 3	71	78	52	0.995	7.40	79	78	60	0.993	8.17
Day 7	69	64	53	0.995	8.10	72	75	51	0.993	5.23
Day 21	74	69	52	0.994	6.18	77	83	57	0.993	7.15
Abomasum Day 1	40	57	27	0.996	4.48	53	77	34	0.995	4.40
Day 3	111	119	73	0.990	3.32	35	39	28	0.997	5.08
Day 7	71	67	55	0.995	6.46	141	188	106	0.985	5.94
Day 21	103	127	79	0.990	8.92	109	144	74	0.992	7.34
Duodenum Day 1	155	191	107	0.982	12.40	120	107	66	0.991	2.04
Day 3	—	—	—	—	—	140	135	118	0.991	34.78
Day 7	231	314	136	0.976	17.85	112	118	91	0.992	13.35
Day 21	—	—	—	—	—	161	160	145	0.991	45.28
Jejunum (proximal) Day 1	277	283	227	0.976	43.97	89	94	73	0.993	3.06
Day 3	410	397	301	0.962	69.11	107	105	83	0.991	8.05
Day 7	315	318	273	0.975	33.62	74	75	64	0.995	3.89
Day 21	76	82	68	0.994	7.66	95	99	83	0.993	8.73
Jejunum (medial) Day 1	184	224	121	0.981	8.92	53	55	42	0.996	1.60
Day 3	212	262	145	0.977	10.26	68	65	57	0.996	1.99
Day 7	—	—	—	—	—	51	52	43	0.996	1.56
Day 21	256	295	163	0.974	16.68	78	79	64	0.994	2.74
Jejunum (distal) Day 1	49	67	35	0.994	2.47	81	76	63	0.994	2.61
Day 3	131	114	99	0.992	7.85	67	64	55	0.995	2.22
Day 7	100	95	82	0.993	5.36	52	53	44	0.996	1.67
Day 21	82	83	73	0.994	6.20	58	63	49	0.996	1.53
Ileum Day 1						75	70	64	0.996	6.74
Day 3	52	55	26	0.995	2.75	93	100	71	0.992	6.33
Day 7	189	197	130	0.981	11.53	77	75	65	0.995	2.19
Day 21	—	—	—	—	—	47	49	41	0.996	1.50
Colon Day 1	—	—	—	—	—	222	217	171	0.981	24.35
Day 3	—	—	—	—	—	129	139	90	0.988	6.33
Day 7	—	—	—	—	—	130	138	104	0.991	9.12
Day 21	—	—	—	—	—	—	—	—	—	—
Fecal Day 0	165	166	134	0.986	14.86	n/a				
Day 1	80	119	44	0.992	3.35	n/a				
Day 2	91	107	62	0.992	8.91	n/a				
Day 3	87	86	67	0.993	12.70	n/a				
Day 4	83	90	71	0.993	8.41	n/a				
Day 5	100	98	83	0.994	16.48	n/a				
Day 7	89	94	80	0.995	15.58	n/a				
Day 14	94	97	71	0.993	6.91	n/a				
Day 21	106	124	49	0.992	4.35	n/a				

### General Features of the Neonatal Calf Microbiome

Measures of α-diversity varied with calf age, anatomical location, and between samples of the lumen and mucosa (Table 1). Richness and α-diversity in luminal samples increased with age in most GIT locations, however, the omasum and distal jejunum exhibited little change between days 3–21 and diversity of the proximal jejunum decreased over the same period (Table 1). By contrast, diversity of mucosal scrapings exhibited no consistent trends and often varied little at each site throughout the trial period (Table 1). Predicted (ACE and Chao1) and observed richness was generally greater in luminal samples of the small intestine (80% of paired sample comparisons for all tests), but in mucosal samples of all other locations (>77% of all paired comparisons) (Table 1). However, α-diversity (the combined measure of richness and evenness) was generally greater in luminal samples at all GIT locations (>66% of age comparisons) (Table 1) suggesting skewed distributions of microbes in mucosal locations. The calf jejunum exhibited the greatest observed and predicted OTU richness and α-diversity across all ages in luminal samples, while the colon initially exhibited the most richness of the mucosal microbiomes before richness of the abomasum (day 7) and duodenum (day 21) increased (Table 1).

**Figure 1:**
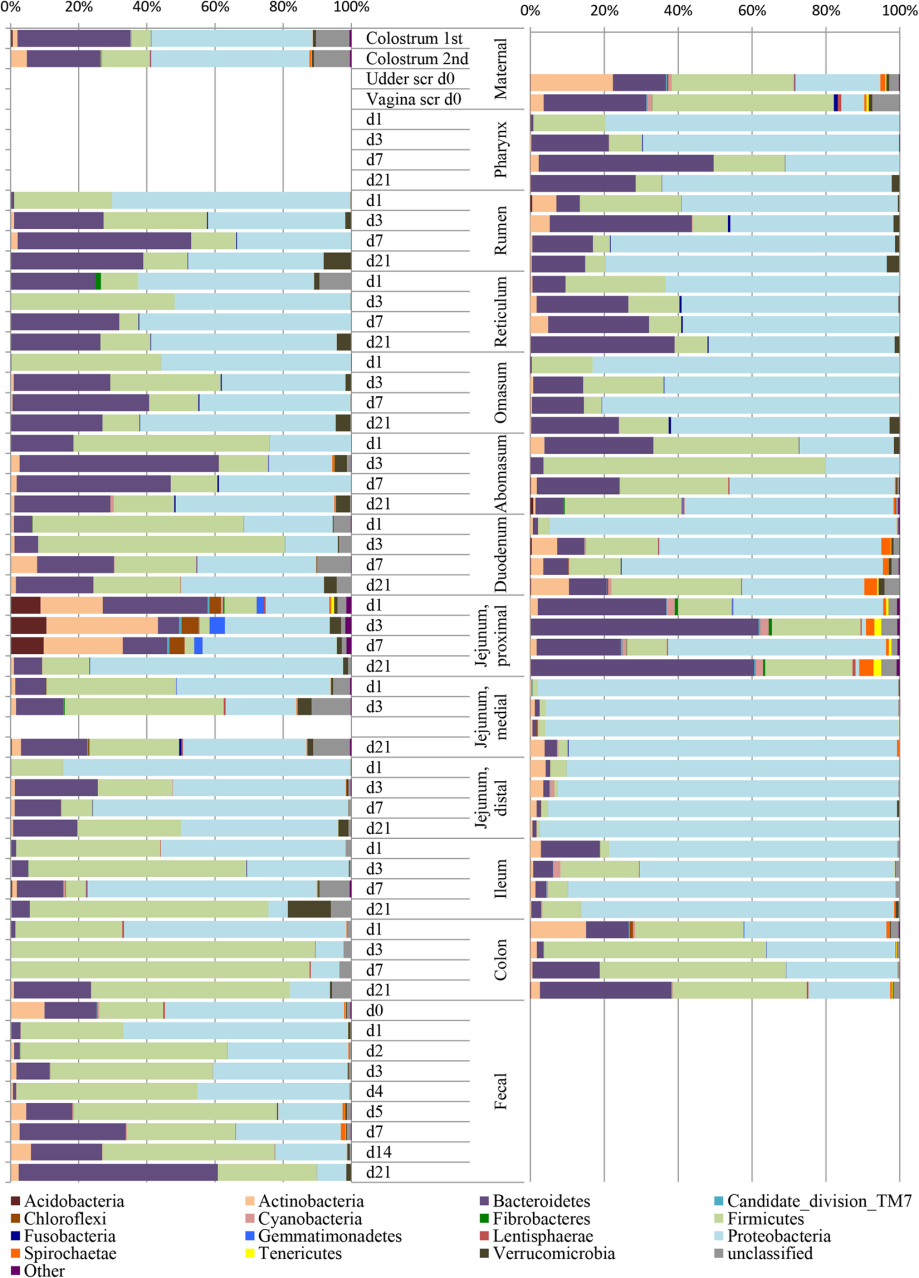
Mean bacterial diversity at the phylum level for maternal and calf lumen (**A**) and mucosal (**B**) samples.

**Figure 2:**
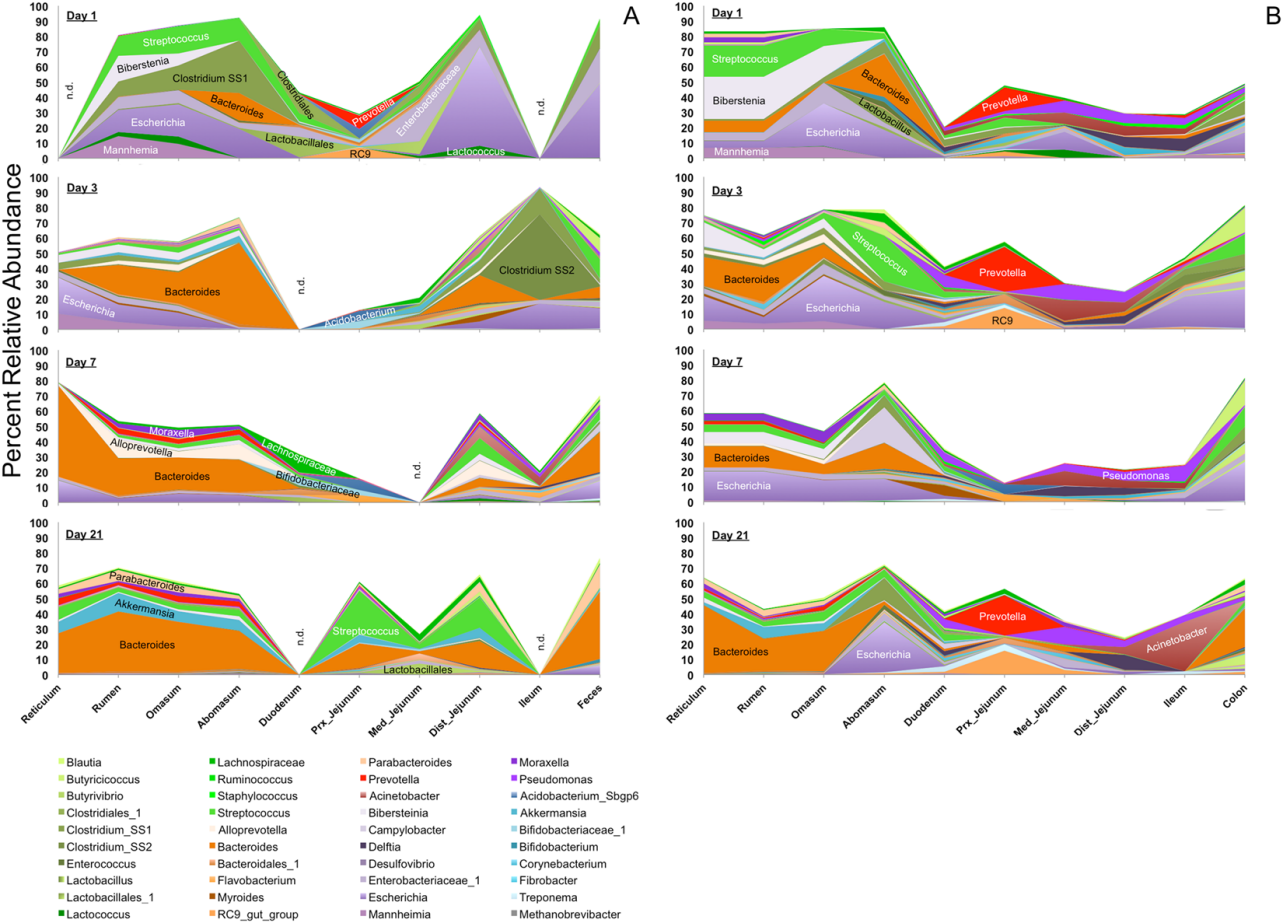
Biogeographical Distribution of Microbial Genera. The percent abundances of the major microbial genera distributed throughout the luminal (**A**) and mucosal (**B**) regions of each GIT location are shown for each age group. Genera are color coded as shown in the figure key as shades of green (Firmicutes), Orange-red (Bacteroidetes), purple (Proteobacteria), blue (other phyla), or grey (Euryarcheota).

Proteobacteria (avg. 41%), Firmicutes (avg. 29%), and Bacteroidetes (avg. 23%) were the most prevalent phyla throughout the early calf GIT (Fig. 1). Proteobacteria had higher relative abundances (on avg. 42% higher) in mucosal samples compared to co-located luminal samples (*P* = 7 × 10^−6^; Fig. S1). Conversely, Firmicutes had higher relative abundances (on avg. 62% higher) in luminal content relative to co-located samples of the mucosa (*P* = 3 × 10^−7^). Firmicutes were also found to have greater relative abundances in the colon (avg. relative abundance = 55%) and feces (avg. 41%) than other GIT locations (*P* < 0.05). Bacteroidetes conversely had greater relative abundances in the reticulum (avg. 26%), rumen (avg. 26%), omasum (avg. 21%), and abomasum (avg. 25%) than in either the colon (avg. 13%) or feces (avg. 13%) (*P* < 0.05). Throughout the GIT, Bacteroidetes were predominantly of the genera *Bacteroides* (avg. 9%) and *Prevotella* (avg. 2%) although the relative abundances of these genera varied with GIT location and between luminal and mucosal microbiota averaging 18% and 20% in mucosal samples of the reticulum and proximal jejunum, respectively (Fig. 2). Consistently, *Bacteroides* spp. had the greatest relative abundance of all genera in the rumen (avg. 17% in lumen and 10% in mucosa), and reticulum (avg. 12% in lumen), and in the luminal content of the omasum (avg. 10%), and abomasum (avg. 15%). *Escherichia* spp. outnumbered *Bacteroides* spp. in the omasum (avg. 14%) and abomasum (avg. 21%) mucosa (Fig. 2). *Lactobacillus* spp. were found to be the most abundant genus in the lumen of the duodenum (avg. 2.7%), being outnumbered by *Pseudomonas* spp. in the mucosa (avg. 5.5%) (Fig. 2). *Pseudomonas*, *Acinetobacter*, and *Delfetia* spp. all had high relative abundances in the mucosal samples of the medial and distal jejunum (avg. 6.9%, 6.9%, and 4.5%, respectively), but were outnumbered by *Prevotella* and the *Rikenellaceae* RC9 group (avg. 10.1%) in the proximal jejunum mucosa. *Streptococcus* spp., which had similar relative abundances in the duodenum (avg. 2.7% in lumen and 3.1% in mucosa), had the highest relative abundance in the lumen of the proximal (avg. 3%) and medial jejunum (avg. 1.7%) and colon (avg. 6.9%) (Fig. 2). *Escherichia* spp. had the highest relative abundance in the lumen of the distal jejunum (avg. 19.4%), in both the lumen (avg. 2.8%) and mucosa (avg. 9.8%) of the ileum, the mucosa of the colon (avg. 15.2%), as well as in feces (avg. 16.9%) (Fig. 2). Pharyngeal samples were predominated by *Biberstenia* spp. (avg. 30.5%) (Fig. 2).

**Figure 3:**
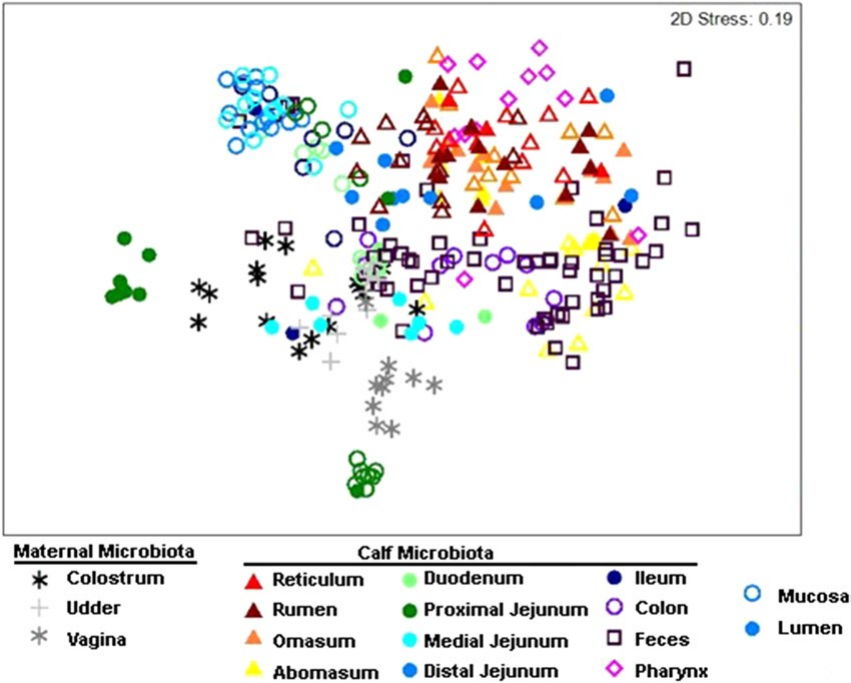
Non-metric multidimensional scaling (nMDS) plot of Bray-Curtis Relationships. Samples are colored by anatomical location, and shaded to reflect mucosal (open) and luminal (closed) samples Maternal microbiota are shown with an asterisk.

Multiple measures of β-diversity (including phylodiversity) indicated the strongest differences to be among GIT locations (PERMANOVA pseudo F = 7.99 *P* = 0.001), and with calf age (pseudo F = 4.25 *P* = 0.001). Luminal and mucosal samples were only different when nested within each GIT location (pseudo F = 4.21 *P* = 0.001), although a weighted UniFraq supported overall differences between the two sample types (Table 2). Further analysis revealed few OTUs were shared between luminal and mucosal samples at each anatomical location (Fig. S2). A non-metric multidimensional scaling (nMDS) of Bray-Curtis dissimilarity revealed potential relationships among various GIT locations (Fig. 3). Notably, abomasal samples largely overlapped with colon and fecal samples, while pharyngeal samples clustered separately. Consistent with these observations, pairwise comparisons of β-diversity for each GIT location revealed no significant differences between the abomasum and either the colon or feces (ANOSIM R < 0.04, *P* > 0.2), or between the colon and feces (ANOSIM R = −0.15, *P* = 0.99). Other relationships were determined among anatomically connected locations, including the the rumen, reticulum and omasum (ANOSIM R < −0.003, *P* > 0.3), and the ileum and each portion of the jejunum (ANOSIM R < −0.06, *P* > 0.1). The ileum was most similar to the distal jejunum and progressively less similar to the medial and proximal jejunum. All other GIT regions differed significantly.

**Table 2:** Multivariate interactions by sample type (Lumen or Mucosa), anatomical location, of age (1, 3, 7, or 21 days), using AMOVA, PERMANOVA, ANOSIM, and Weighted UniFrac.

Interaction	AMOVA	PERMANOVA			ANOSIM		Weighted UniFrac	
	P	Df	Pseudo-F	P	R	P	W score	P
Anatomical Location (L)	<0.03^A,B^	13	7.99	0.001	0.09–0.048^C^	<0.05	0.84^B^	<0.002^B^
Sample Type (T)	ns			ns	0.06	<0.001	0.56	<0.001
Age (A)	All: <0.05 Lumen: <0.001 Mucosa: <0.001, for d1 X d21	7	4.25	0.001	0.07–0.29	<0.05	0.78	<0.004
T x A	<0.05	3	2.25	0.001	0.18	<0.001	0.80	<0.001
L x T	ns	8	4.21	0.001	0.35	<0.001	0.86	<0.001
L x A	All: ns Lumen: <0.05, for omasum Mucosa: ns	33	1.53	0.001	0.53	<0.001	0.87	<0.001
T x L x A	ns	18	1.19	0.021	0.60	<0.001	0.91	<0.001

^A^Non-significant comparisons: abomasum/colon/fecal, duodenum/ileum, distal jejunum/ileum.

^B^Non-significant comparisons: rumen/omasum/reticulum, duodenum/jejunum medial, jejunum medial/jejunum distal.

^C^Significant comparisons: proximal jejunum/medial jejunum/colon, proximal jejunum/abomasum, colon/omasum, duodenum/distal jejunum, fecal/reticulum, duodenum/fecal, fecal/omasum, abomasum/reticulum, abomasum/rumen, pharynx/rumen, omasum/pharynx, pharynx/reticulum.

Linear discriminant analyses revealed an increasing number of taxa characterizing each age group, with samples collected from 21 day old calves containing the largest number of discriminatory OTUs (OTUs significantly delineating each group) including the only *Prevotella* OTU detected (A second OTU whose taxonomy could only be resolved to the family Prevotellaceae may also have been a *Prevotella* spp.; Table 3).

**Table 3:** Linear Discriminant Analysis values by sample day. Only OTUs with statistically significant LDA scores are presented, p-values obtained by Wilcox Rank test and were all <0.05.

Taxon	LDA	Taxon	LDA
Sampling Day 1			
Enterobacteriaceae	4.51205	*Enterobacter*	2.87377
Bacilli	3.07302		
Sampling Day 3			
*Veillonella*	4.36347	Veillonellaceae	3.83645
Clostridiaceae	4.04293	*Acinetobacter*	3.7281
Pasteurellaceae	4.01357	Erysipelotrichaceae	2.63438
Sampling Day 7			
*Porphyromonas*	4.16	*Flavonifractor*	3.11288
*Alloprevotella*	3.96851	*Stenotrophomonas*	3.07257
*Trueperella*	3.49458		
Sampling Day 21			
*Prevotella*	4.06042	Neisseriaceae	3.64117
*Akkermansia*	4.00454	vadinBB60	3.3566
*Parabacteroides*	3.96574	BS11_gut_group	3.33279
*Gallibacterium*	3.90873		

### Maternal Sources of Microbiota

The mean α-diversity indices for maternal sources of microbiota indicated the udder skin had the greatest richness and diversity, followed by colostrum, and then vaginal samples (Table 1). Measures of α-diversity did not significantly differ between colostrum samples when immediately collected and following refrigeration for 4 h. Colostrum and udder skin samples differed significantly (PERMANOVA pseudo F = 4.98, *P* < 0.001) sharing just 11 OTUs (<7% of observed). Both colostrum and udder skin samples exhibited even greater dissimilarity when compared to vaginal samples (pseudo F > 9.9, *P* < 0.001).

Colostrum was determined to predominantly harbor bacteria of the phyla Proteobacteria (avg. 42%), Firmicutes (avg. 22%), and Bacteroidetes (avg. 21%) (Fig. 1). The genus *Lactococcus* was found to have the greatest relative abundance (average 13.6%) in colostrum prior to refrigeration, however, its relative abundance decreased to less than 0.5% in all colostrum samples following refrigeration. Similar changes were seen in *Staphylococcus* spp. (avg. 1.3% to 0.12%). The decreased relative abundances of these genera following refrigeration corresponded to 1.5–2 fold increases in numerous, mostly unclassified genera of the Proteobacterial phylum. The genera *Pseudomonas* (avg. 2.3%), *Bacteroides* (avg. 1.4%), *Streptococcus* (avg. 1.3%), *Bifidobacterium* (avg. 0.6%), *Akkermansia* (avg. 0.4%), *Escherichia* (avg. 0.3%), and *Lactobacillus* (avg. 0.2%) were also prevalent along with unresolvable members of the bifidobacteriaceae family (avg. 0.1%) but appeared to be unaffected by refrigeration.

Samples of the dam’s udder skin and vagina were similarly determined to predominantly harbor the phyla Firmicutes (avg. 34% and 49%, respectively), Bacteroidetes (avg. 15% and 28%, respectively), Actinobacteria (avg. 23% and 4%, respectively) and Proteobacteria (avg. 20% and 6%, respectively) (Fig. 1). The genera with the highest relative abundances on the udder skin comprised a mix of common host commensal and environmental bacteria that are typically aerobic or facultatively anaerobic including *Corynebacterium* (avg. 5.2%), *Arthrobacter* (avg. 4.9%), *Pseudomonas* (avg. 4.1%), *Streptococcus* (avg. 3.3%), *Acinetobacter* (avg. 2.2%) and *Staphylococcus* (avg. 2.0%) (Fig. 2). The vagina was instead found to have high abundances of many genera commonly described in the adult rumen, including *Bacteroides* (avg. 5.2%), the Rikenellaceae RC9 group (avg. 4.9%), *Ruminococcus* (avg. 1.1%), and *Butyrivibrio* (avg. 1.1%) (Fig. 2). Additionally, with the exception of a single udder skin sample, the vagina was the only maternal source where archaea (i.e. methanogens) were detected, being observed in seven of ten vaginal scrape samples. Linear discriminant analysis (LDA) concluded that udder skin and vaginal samples had the largest numbers of discriminatory OTUs among maternal sources examined (Table 4). Discriminatory OTUs of the vagina included additional well-described members of ruminant GIT microbiota including OTUs that classified as *Ruminococcus*, *Prevotellaceae*, and *Pseudobutyrivibrio*.

**Table 4:** Linear Discriminant Analysis values by anatomical location from maternal and calf sources. Only OTUs with statistically significant LDA scores are presented, p-values obtained by Wilcox Rank test and were all <0.05.

Taxon	LDA	Taxon	LDA	Taxon	LDA
**Maternal**					
**Colostrum**					
Bacteroidetes	4.35	RFP12_gut_group	3.31	*Citrobacter*	2.92
Alphaproteobacteria	4.27	Caulobacteraceae	3.28	*Woodsholea*	2.86
*Azorhizophilus*	3.98	Sphingomonadaceae	3.21	*Falsirhodobacter*	2.82
Gammaproteobacteria	3.84	Flavobacteriales	3.15	Sphingobacteriales	2.76
Methylococcales	3.49	*Caulobacter*	3.03	Rhodocyclaceae	2.70
Xanthomonadaceae	3.36				
**Udder Skin**					
*Corynebacterium*	4.39	*Arcanobacterium*	3.38	*Aequorivita*	2.94
*Arthrobacter*	4.32	*Trichococcus*	3.33	*Ornithobacterium*	2.94
*Staphylococcus*	3.99	*Atopostipes*	3.32	Clostridiales	2.91
Micrococcaceae	3.87	Staphylococcaceae	3.26	*Peptoniphilus*	2.89
*Ornithinimicrobium*	3.87	Corynebacteriaceae	3.22	*Brevibacterium*	2.81
*Bifidobacterium*	3.66	*Solibacillus*	3.17	*Alloiococcus*	2.74
*Dietzia*	3.62	*Murdochiella*	3.14	*Petrimonas*	2.71
Micrococcales	3.61	*Janibacter*	3.14	*Caryophanon*	2.66
Intrasporangiaceae	3.61	Corynebacteriales	3.10	*Enteractinococcus*	2.65
*Jeotgalicoccus*	3.55	*Luteimonas*	3.02	*Salinicoccus*	2.59
*Chryseobacterium*	3.53	*Eremococcus*	3.01	*Defluviimonas*	2.51
Planococcaceae	3.47	*Aerococcus*	2.99	Epsilonproteobacteria	2.47
*Ornithinicoccus*	3.40	*Fastidiosipila*	2.96	*Turicella*	2.40
**Vagina**					
Ruminococcaceae	4.88	vadinBB60	3.53	*Anaerovorax*	2.94
Bacteroidales	4.37	Clostridia	3.53	*Barnesiella*	2.93
Lachnospiraceae	4.29	*Desulfovibrio*	3.51	*Geobacillus*	2.92
Christensenellaceae	4.23	*Sporobacter*	3.45	*Sutterella*	2.89
Ruminococcaceae	4.15	*Victivallis*	3.39	*Acetitomaculum*	2.81
Peptostreptococcaceae	4.13	Defluviitaleaceae	3.39	NB1-n	2.79
Clostridiales	4.09	*Anaerotruncus*	3.33	M2PT2-76_termite_group	2.76
*Alistipes*	4.07	Clostridiales	3.31	*Aeriscardovia*	2.74
*Phocaeicola*	3.92	Prevotellaceae	3.31	Oscillospira	2.70
Gastranaerophilales	3.80	*Ruminobacter*	3.27	*Odoribacter*	2.68
*Ruminococcus*	3.74	*Thalassospira*	3.27	*Syntrophococcus*	2.68
dgA-11_gut_group	3.71	*Paraprevotella*	3.25	*Thermoactinomyces*	2.66
*Dorea*	3.70	*Pseudobutyrivibrio*	3.25	*Hydrogenoanaerobacterium*	2.64
*Rikenellaceae*	3.70	*Intestinimonas*	3.24	*Planifilum*	2.63
*Butyrivibrio*	3.70	*Paludibacter*	3.24	BG.g7	2.60
*Phascolarctobacterium*	3.67	Coriobacteriaceae	3.18	*Christensenella*	2.60
Clostridiales	3.54	Desulfovibrionaceae	3.14		
**Calf**					
**Pharynx**					
*Bibersteinia*	5.18	*Flavonifractor*	3.69		
**Rumen**			**Duodenum**		
*Alloprevotella*	4.09			Rhodospirillaceae	2.57
**Jejunum (proximal)**					
*Succiniclasticum*	3.28	Oxalobacteraceae	3.02	*Oscillibacter*	2.82
**Jejunum (medial)**			**colon**		
Enterobacteriaceae	4.4			*Weeksella*	3.2

### Early Succession of the Neonatal Calf Microbiome and Maternal Influences

**Figure 4:**
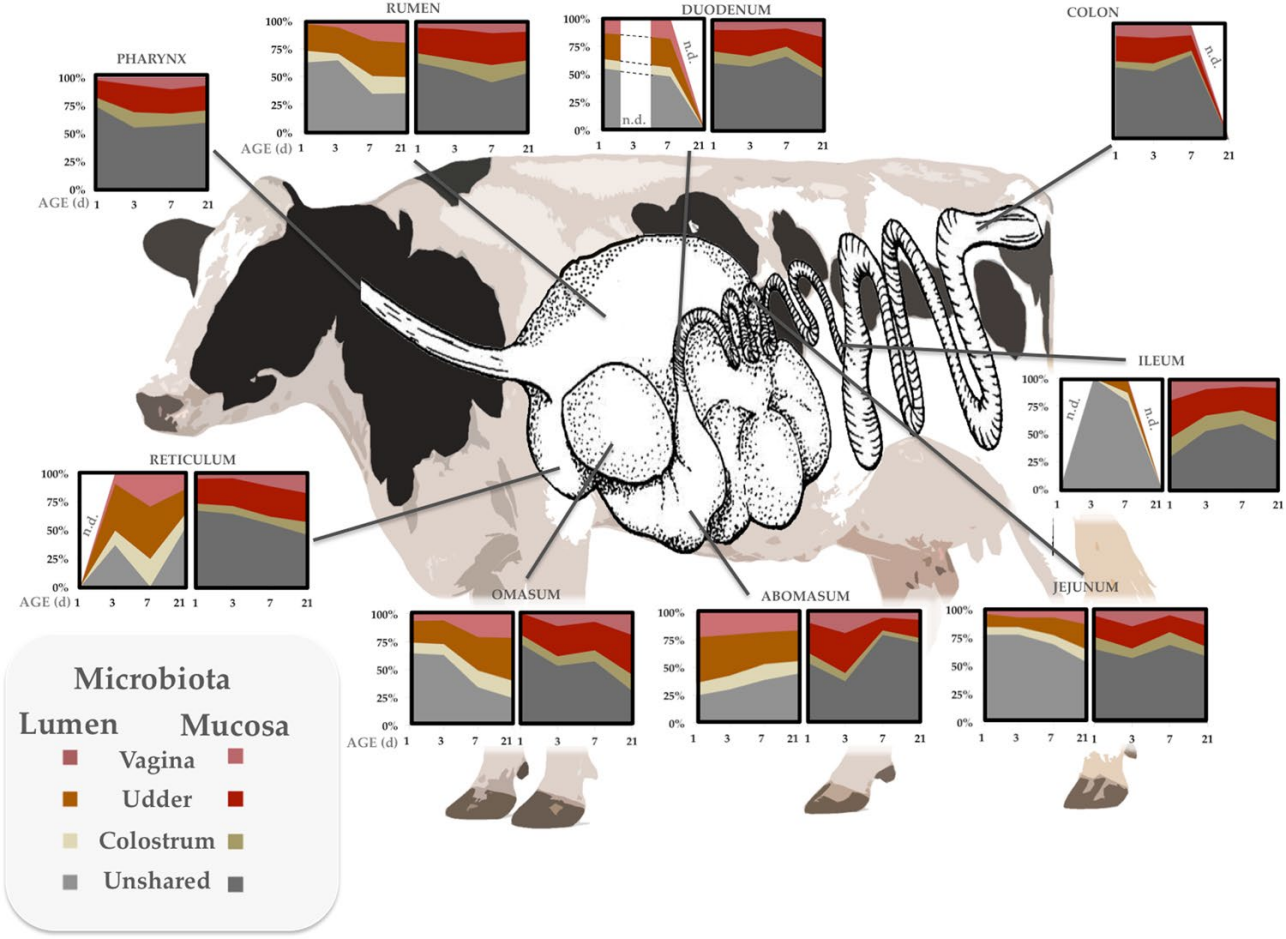
Proportion of OTUs shared with maternal sources across GIT locations and between mucosa and lumen with age. Jejunum proportions are averages of the proximal, medial, and distal region.

Overall 46.1 ± 22% of luminal and 41.4 ± 12% of mucosal OTUs seen throughout the calf GIT were present in one or other maternal source (Fig. 4). Generally, the numbers of OTUs shared between maternal sources and the calf GIT increased for each GIT location over the first 21 days of life in both luminal and mucosal regions, with exception of the abomasum, which instead decreased (Fig. 4). The udder skin microbiota shared the largest number of OTUs with both luminal (avg. 23.9%) and mucosal (avg. 21.8%) microbiota of the calves, sharing approximately twice the number of OTUs as either colostrum (avg. 10.6% in lumen, and 9.6% in mucosa) or vaginal (avg. 12.5% and 10%, respectively) samples overall and at all ages from day 1–21 (Fig. 3). This was also true for all regions of the GIT except the mucosa of the proximal jejunum, which shared more OTUs with vaginal microbiota at days 3 (avg. 20.6% vs. 36.2%, respectively) and 21 (avg. 21.7% vs. 38.6%, respectively). While the number of OTUs shared with the udder skin increased over the first week for luminal samples (excluding the abomasum), numbers shared by mucosal samples did not vary substantially (Fig. 4).

Because OTUs shared between the different calf GIT regions and the maternal sources varied in their abundances, measures of β–diversity indicated that, although all calf GIT and fecal samples were significantly different from each maternal reservoir (ANOSIM R > 0.2, *P* < 0.01), several had greater or equivalent Bray-Curtis (BC) similarity to maternal colostrum or vaginal samples (Fig. S3). This included luminal samples of the rumen, reticulum, and omasum which exhibited the least BC similarity to vaginal samples (*P* < 0.05) among all maternal sources at day 1, with the later two GIT regions being initially more similar to colostrum (*P* < 0.05), but by day 21 all had equivalent BC similarity to the vagina and udder skin and greater BC similarity than colostrum (*P* < 0.05). With the exception of the proximal and distal jejunum, the ileum, and the colon all mucosal samples did exhibit the greatest BC similarity to maternal udder skin samples by day 21, although the reticulum and omasum had equivalent BC similarity to maternal vaginal samples. Instead the microbiota of the proximal jejunum were more similar to those of the dam’s vagina at days 1, 3 and 21, and the distal jejunum was more similar to colostrum samples at all ages. The ileum was initially more similar to maternal udder skin samples but by day 7 had identical BC similarity to second-fed colostrum and was more similar to first-fed colostrum at day 21 (*P* < 0.05). The colonic mucosa was initially most similar to the maternal udder skin, but became most similar to second-fed colostrum at days 3 and 7 before becoming more similar to the dam’s vagina at day 21 (*P* < 0.05) and this pattern was largely reflected in fecal samples.

Ewans’-Caswell^42^ analyses revealed the calf GIT microbiota deviated little from the assumptions of neutral theory at all anatomical locations from age 1–21d (Fig. S4). Each GIT location exhibited less deviation from the assumptions of neutral theory than seen in colostrum (*P* < *0*.*05*), the deviation statistic (*V*) of which indicated greater dominance by select taxa than would be expected in a neutral model. Of maternal sources, colostrum exhibited the largest deviation from the assumptions of neutral theory (*P* < 0.05), and the udder skin exhibited the least numerically, though was not statistically different from the vagina (*P* = 0.23). The udder skin was not significantly less neutral than any GIT region, and the vagina was only different from the reticulum and omasum (*P* < 0.05), although trended toward a difference from the rumen (*P* = 0.09).

## Discussion

Several studies have previously described the microbiota of the cattle vagina^56–60^, skin^61^, and colostrum^62^. The microbiota determined in our study are each consistent with those reported previously.

In our study, vaginal microbiota were not found to be dominated by *Aggregatibacter* or *Streptobacillus* spp., as previously described by Swartz *et al*.^56^, however, many of the other genera described in that study, and most described in separate studies of Nellore cattle by Laguardia-Nascimento *et al*.^57^ were observed. These included *Myroides*, *Flavobacterium*, *Lactobacillus*, *Campylobacter*, and *Escherichia* spp., as described by Swartz and colleagues^56^ and *Alistipes*, *Bacillus*, *Ruminococcus*, *Rikenella*, and *Prevotella* spp. described by Laguardia-Nascimento and colleagues^57^. Both studies have now reported *Bacteroides*, and methanogenic archaeal populations in the cattle vagina^56,57^. These microbial groups have also been recently detected in the uterus of dairy cattle^63,64^. A fourth study investigating the vaginal microbiota associated with bovine necrotic vulvovaginitis^58^ only reported phylum-level classifications. In that study the authors described: i) *Bacteroidetes* and *Proteobacteria* levels consistent with this study and the studies of Swartz *et al*., and Laguardia-Nascimento and colleagues^56,57^; ii) *Firmicutes* at levels consistent with this study and that of Laguardia-Nascimento and colleagues^57^; and iii) *Fusobacteria* levels consistent with that of Swartz and colleagues^56^.

Microbiota of the udder skin, were also consistent with a previous survey of cattle teat skin microbiota^61^ that used both cultivation and culture-independent techniques and reported *Corynebacterium* spp. (specifically, *C*. *freneyi*, *C*. *pilosum*, *C*. *vitaeruminis*, *C*. *xerosis* and other *C*. *spp*.) and *Staphylococcus* spp. (specifically *S*. *arlettae*, *S*. *fleurettii*, *S*. *haemolyticus*, *S*. *pasteuri*, *S*. *saprophyticus*, *S*. *succinus*, *S*. *vitulinus*, *S*. *xylosus* and other *S*. spp.) to be abundant along with lower abundances of *Arthrobacter* (*A*. *gandavensis*), *Pseudomonas* (*P*. *sp*.), and *Streptococcus* (*S*. *equinus*) spp.

Likewise, the colostrum microbiota determined in this study were similar to those previously reported in cattle^32,62^, as well as to a more recent study in goats^31^. Both^31^ and^32^ reported *Lactococcus* (Ruiz *et al*. specifically identified *L*. *lactis*) and *Lactobacillus* (Lindner *et al*. identified *L*. *casei*, and Ruiz *et al*. identified *L*. *delbrueckii* and *L*. *fermentum*), while all three studies identified *Staphylococcus* (Ruiz *et al*. identified *S*. *pseudintermedius* and *S*. *chromogenes*)^31,32,62^. Both Ruiz *et al*.^31^ and^62^ also reported *Escherichia* spp. to be prevalent. *Lactococcus*, the most abundant genera identified in this study, is also commonly found in human colostrum^30^, and antibodies against *Lactococcus* phages have previously been noted in cow colostrum^65^.

The entire calf GIT microbiota appeared to be influenced by microbes of the dam’s vagina, udder skin, and colostrum. All three maternal sources comprised a unique microbial reservoir that shared OTUs with all examined calf GIT locations and in both luminal and mucosal subsamples. These shared OTUs were detected in the earliest samples and persisted through the 21 day sampling period. The udder skin shared the greatest number of OTUs with almost all regions of the calf GIT at each time point. Because calves had no contact with the dam following their separation at birth, when these samples were collected, this relationship between the GIT and udder skin microbiota cannot be reflective of microbes transferred in the opposing direction from the calves oral microbiota to the teat and surrounding skin surface. However, the finding that the udder skin exhibited little deviation from the assumptions of neutral theory (that the community has developed stochastically from non-interacting populations and may persist temporarily^42^) may indicate that the sample also reflects allocthonous microbiota picked up from the environment in addition to any autochthonous skin microbiota. We do, however, note that the major genera identified on the udder skin in this study are well documented among skin microbiota of cattle^61^ and other mammalian species^66^ where they have been shown to exhibit temporal stability^66^. Though it is noteworthy that many are dually observed on skin and in the environment (e.g. soil and water), including *Arthrobacter*, *Pseudomonas*^67^, *Streptococcus*^68^, *Acinetobacter*^69,70^, and *Corynebacterium* and may be passed between host and environmental niches^71,72^. It is therefore plausible that many or all of the microbes shared between the udder skin and calf GIT were not maternally transferred but instead seeded directly or indirectly, perhaps even continuously from the environment.

This is less likely to be true of vaginal or colostrum microbes. The vaginal microbiota are likely to represent the first source of microbes due to the order in which the calf is exposed to each of these maternal sources. Despite the brevity of this interaction, the vagina appears to have a profound influence contributing many microbial taxa with well-described physiological roles in the adult rumen. This includes a nearly unique reservoir of methanogenic archaea among the maternal sources profiled in this study. Methanogenic archaea, alike many of the bacterial genera observed in the vagina are strict anaerobes^73^ and are unlikely to viably persist in the environment for any significant period of time. Therefore, they’re transmission is most likely to occur through direct maternal interactions. The anatomical orientation of the anus above the vagina in cattle may serve to seed the vagina with these common GIT microbes and so it remains to be determined if these archaea and other common rumen microbes truly colonize the vagina or are prevalent due to a sink:source relationship. Marginal deviations from the assumptions of neutral theory may support the later, however, several studies evaluating the potential for fecal contamination of the cow vagina, mostly in the context of disease (e.g. endometritis) do not support this as a typical route of transfer^74^. Nevertheless, the presence of these rumen microbes in the vagina avails an early opportunity to seed the neonatal calf GIT and understanding this relationship may be important to on-going ecological engineering efforts aimed at replacing ruminal methanogenesis with alternative forms of hydrogenotrophy^75^.

Colostrum exhibited the largest deviation from the assumptions of neutral theory and similar to previous reports, was found to possess a unique microbiota from that observed on the surrounding skin^61^, supporting the hypothesis that colostrum possess an autochthonous microbiota. Many of the microbes we detected in colostrum were also observed in the calf GIT, suggesting colostrum contributed to the make-up of the calf GIT. Colostrum is known to be of additional importance to host physiology and microbial ecology because it provides the earliest form of nutrition both directly to the neonate and to the early microbiota^30,76^, along with immunological factors^77^. Consequently, the timely acquisition of high-quality colostrum is a proven factor in sustaining the GIT health of young calves^77^ and it would be interesting to more explicitly examine the nutritive and immunological influences of these colostrum-mediated factors on the early ruminant microbiota.

These were clearly not the only sources of microbes to the neonatal calf GIT as evidenced by the finding that only ~46% of OTUs observed in the calf GIT were detected in one or other maternal sample. Under ordinary circumstances, the dam’s oral microbiota may also be important. This is particularly true given cattle frequently regurgitate feed from their rumen in a process known as rumination^78^, which could facilitate the oral transfer of important rumen microbial groups, similar to those observed in the dam’s vagina. However, because calves were separated from the dam within 15 minutes of parturition, the direct transfer of maternal microbes from the oral cavity was limited. Feed-borne microbes may also be important, although a study by Piao and colleagues^79^ revealed a rapid loss of signal for microbes colonizing switchgrass upon immersion within the rumen. It is also unclear if calves are colonized in utero by a placental or uterine microbiota. Studies have recently revealed microbial signatures in the ruminant uterus^80,81^ and it would be interesting to establish if these microbes are viably transferred to the calf prior to parturition.

Despite little measurable deviation from the assumptions of neutral theory in the calf GIT microbiota, it was found to exhibit biospatial and longitudinal differences over the first 21 days. Only several anatomically connected regions were determined to be inseparable by measures of β-diversity, along with the abomasum, which was seen to be similar to the colon and feces. Two previous studies reporting biospatial variation through the GIT of lambs^16^ and dairy cattle^15^ also found no significant variation between the colon and either feces, or the rectum, respectively. The additional relationship with the abomasum observed in this study may reflect early ruminant GIT physiology where a unique evolutionary feature, the oesophageal groove, that causes ingested feed to bypass the underdeveloped rumino-reticulum and instead empty directly into the abomasum^82^. Perhaps in relation to this, Firmicutes were seen to have significantly greater relative abundances in the colon and conversely Bacteroidetes had significantly greater relative abundances within the bypassed foregut. This delineation is reflective of observations of various animals during and after hibernation where the relative abundance of Bacteroidetes in the GIT has been seen to be greater than Firmicutes when the animal is hibernating, while Firmicutes rapidly outnumber Bacteroidetes following emergence and a return to eating^83,84^. The ratio of Bacteroidetes to Firmicutes could therefore reasonably be hypothesized to reflect the differing nutritional status along the GIT. It is also worth noting that the greater relative abundance of Bacteroidetes in the rumen is inconsistent with studies of the matured adult rumen where Firmicutes are typically shown to have the highest relative abundances in both the rumen and colon^85,86^. Within each GIT location, differences were also seen between luminal and mucosal samples likely reflecting the differing nutritional and physiological conditions in these regions. For example, genera well described as aerobic or facultatively anaerobic including *Escherichia*, *Pseudomonas*, *Acinetobacter*, and *Delftia* were seen to have the greatest relative abundances in many of the mucosal samples and this may reflect their resistance to, need of, or preference for higher oxygen concentrations available nearer the gastrointestinal epithelium^24^. This may also explain why udder skin microbiota almost always exhibited the strongest relationship to the mucosa. As has been described in other studies of microbiota succession, we observed increasing richness and α-diversity with age, and show that this is observable throughout the GIT with the exception of the distal jejunum.

Collectively, we have provided the first description of the maternal influence on the early ecological dynamics of microbiota throughout the calf GIT, revealing immediate biogeographical delineation of these microbiota among each GIT location and between mucosal and luminal regions. We have also uncovered unique and potentially important influences of microbiota from the skin of the dam’s udder, the vagina, and colostrum, which each appear to make early contributions to the biospatial and longitudinal succession of microbes throughout the early calf GIT.

### Ethics, Consent, and Permissions

All experimental procedures were performed at Fair Oaks Farms (Fair Oaks, IN) and University of Illinois Veterinary Medicine Research Farm (Urbana-Champaign, IL) under protocols approved by the Institutional Animal Care and Use Committee (IACUC) of the University of Illinois (protocol #13096).

### Consent for Publication

All authors have read and approved the final version of the manuscript and consent to its publication.

### Availability of data and materials

Raw sequencing data and metadata are available through NCBI under BioProject accession PRJNA314799.

## Electronic supplementary material

Dataset 1
