# Supplementary material for: Biogeographical Differences in the Influence of Maternal Microbial Sources on the Early Successional Development of the Bovine Neonatal Gastrointestinal tract
Source: Sci Rep. 2018 Feb 16;8:3197. doi: 10.1038/s41598-018-21440-8 (PMC5816665; doi:10.1038/s41598-018-21440-8)
Supplement: Supplementary file 1 — Dataset 1 [file 41598_2018_21440_MOESM1_ESM.doc]

**Biogeographical Differences in the Influence of Maternal Microbial Sources on the Early Successional Development of the Bovine Neonatal Gastrointestinal tract.**

Carl J. Yeoman^1*^, Suzanne L. Ishaq^1^, Elena Bichi^2^, Sarah K. Olivo^1^, James Lowe^2^, Brian M. Aldridge^2*^

**Figure S1 Stripplot showing ratios of the relative abundances of the four major phyla in co-located mucosal and luminal samples.**

**
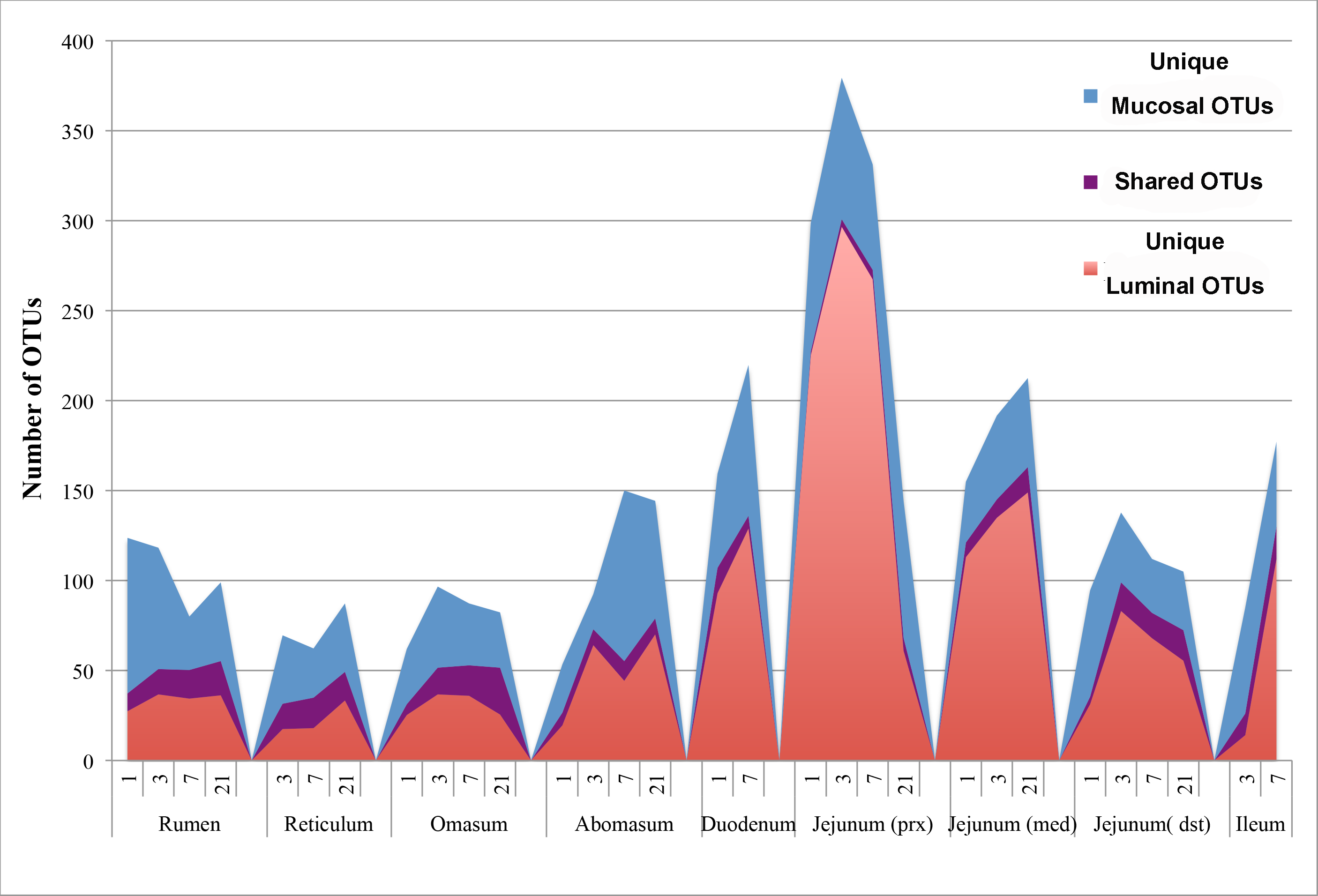
**

**Figure S2 Shared and unique OTUs between luminal and mucosal samples at each GIT location.**


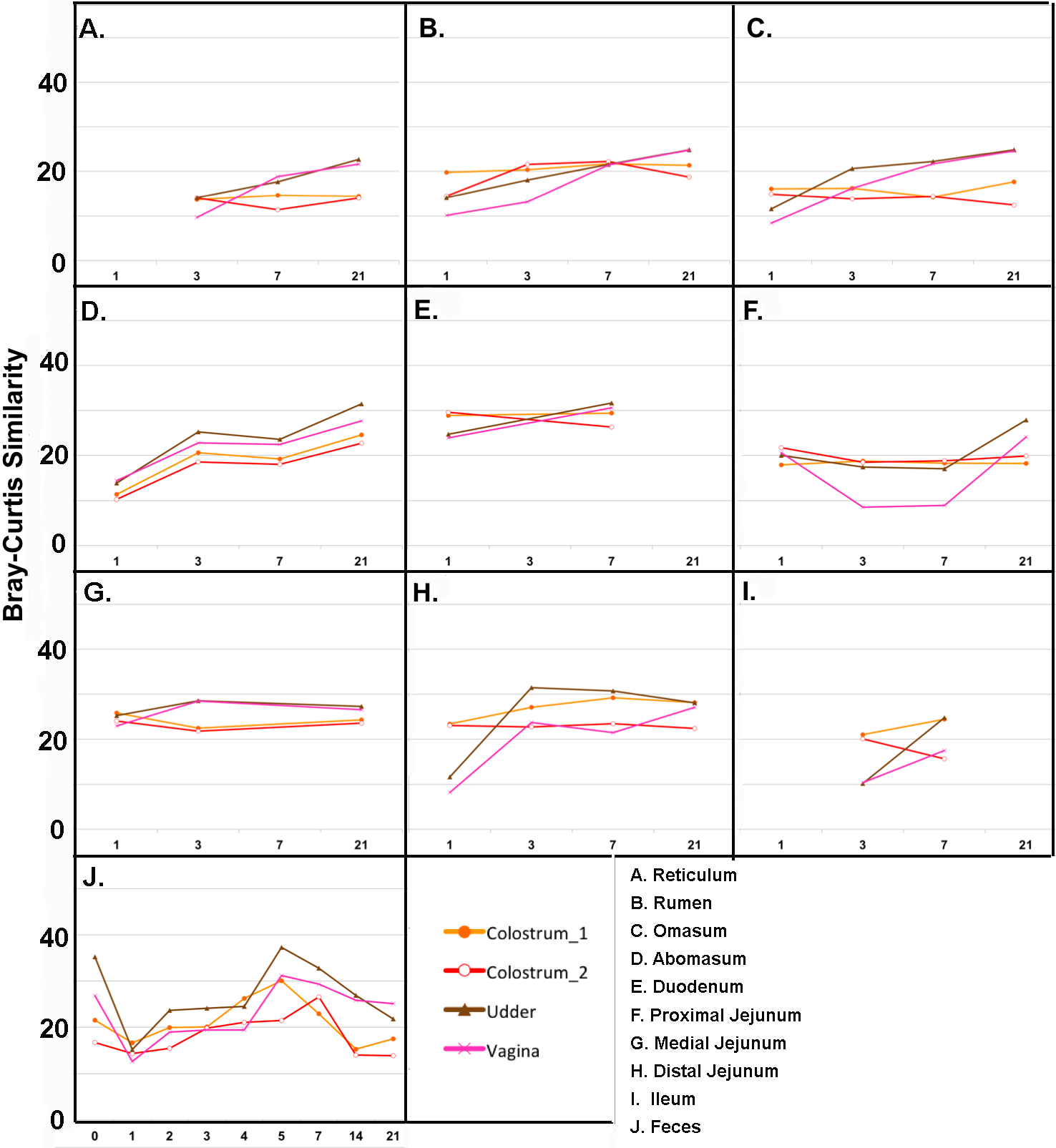


**Figure S3 Bray-Curtis relationships between maternal microbiota and luminal calf microbiota at each GIT location by age.**


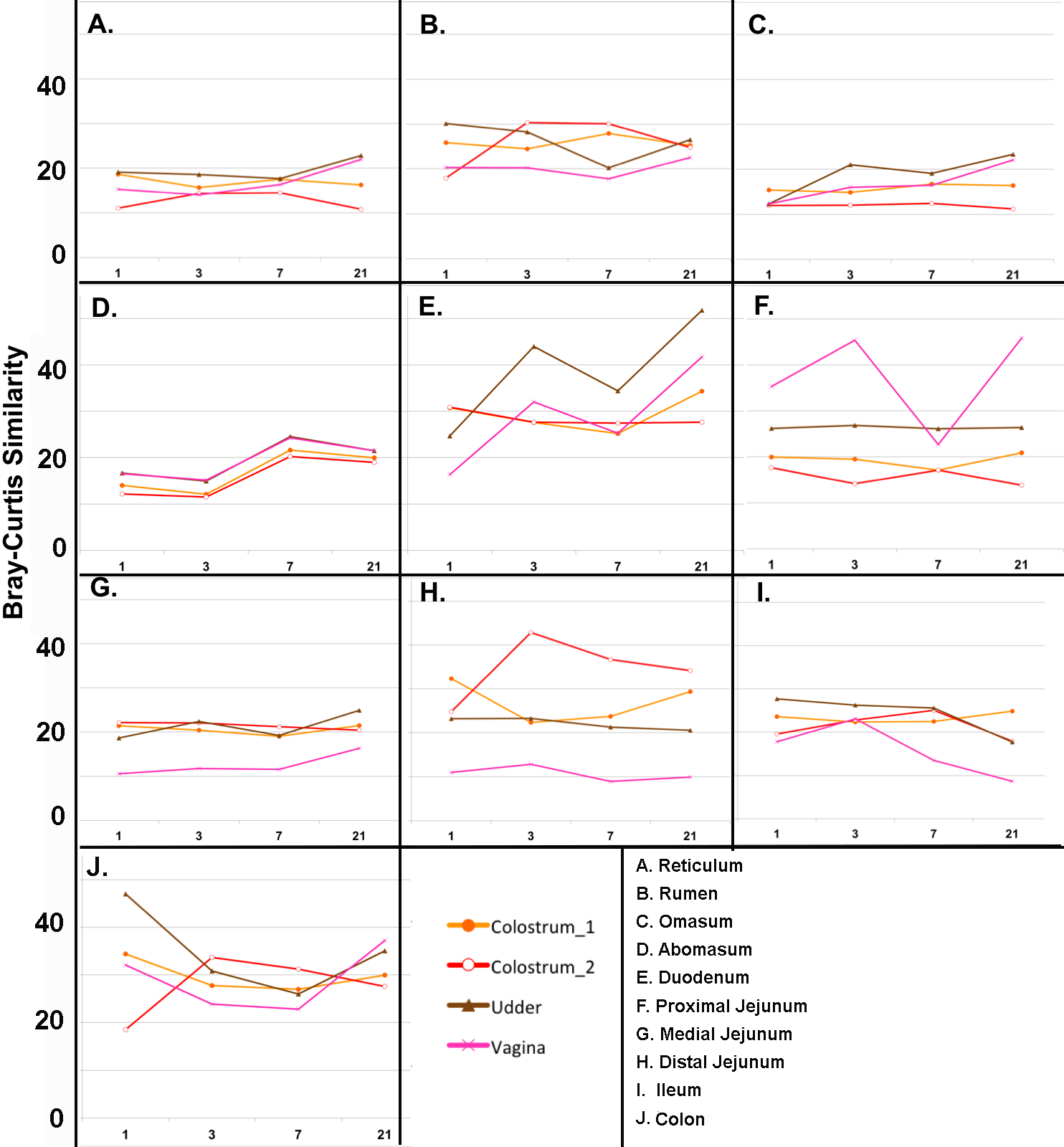


**Figure S4 Bray-Curtis relationships between maternal microbiota and mucosal calf microbiota at each GIT location by age.**


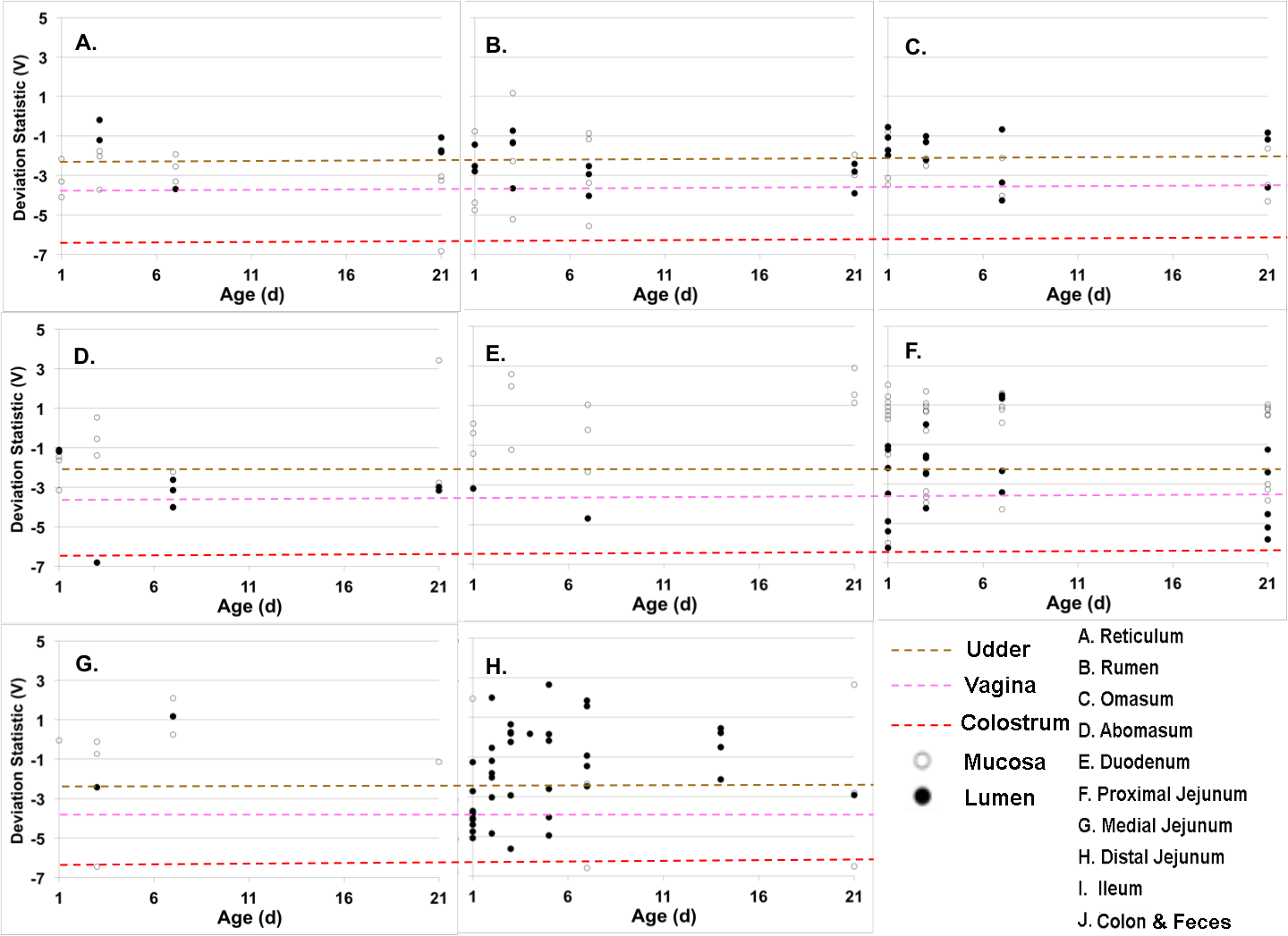


**Figure S5. Ewan’s-Caswell V-statistic for each calf GIT location as compared to maternal sources.**
